# Fabrication and characterization of nanodelivery platform based on chitosan to improve the anticancer outcome of sorafenib in hepatocellular carcinoma

**DOI:** 10.1038/s41598-023-38054-4

**Published:** 2023-07-27

**Authors:** Fahad Albalawi, Mohd Zobir Hussein, Sharida Fakurazi, Mas Jaffri Masarudin

**Affiliations:** 1 Department of Medical Laboratory and Blood Bank, King Fahad Specialist Hospital-Tabuk, Tabuk, Saudi Arabia; 2 Nanomaterials Synthesis and Characterization Laboratory, Institute of Bioscience (IBS), Universiti Putra Malaysia, Serdang, Selangor Malaysia; 3 Faculty of Dentistry, Universitas Brawijaya, Malang, Indonesia; 4 Department of Human Anatomy, Faculty of Medicine and Health Sciences, Universiti Putra Malaysia, Serdang, Selangor Malaysia; 5 Natural Medicine and Product Research Laboratory Institute of Bioscience, Serdang, Selangor Malaysia; 6 Department of Cell and Molecular Biology, Faculty of Biotechnology and Biomolecular Sciences, Universiti Putra Malaysia, Serdang, Selangor Malaysia

**Keywords:** Cancer, Materials science, Nanoscience and technology

## Abstract

Chitosan nanoparticles (CS NPs) showed promising results in drug, vaccine and gene delivery for the treatment of various diseases. The considerable attention towards CS was owning to its outstanding biological properties, however, the main challenge in the application of CS NPs was faced during their size-controlled synthesis. Herein, ionic gelation reaction between CS and sodium tripolyphosphate (TPP), a widely used and safe CS cross-linker for biomedical application, was exploited. The development of nanodelivery platform, namely Sorafenib-loaded chitosan nanoparticles (SF–CS NPs), was constructed in order to improve SF drug delivery to human Hepatocellular Carcinoma (HepG2) cell lines. The NPs were artificially fabricated using an ionic gelation technique. A number of CS NPs that had been loaded with an SF were prepared using different concentrations of sodium tripolyphosphate (TPP). These concentrations were 2.5, 5, 10, and 20 mg/mL, and they are abbreviated as SF–CS NPs 2.5, SF–CS NPs 5.0, SF–CS NPs 10, and SF–CS NPs 20 respectively. DLS, FTIR, XRD, HRTEM, TGA, and FESEM with EDX and TEM were used for the physiochemical characterisation of SF–CS NPs. Both DLS and HRTEM techniques demonstrated that smaller particles were produced when the TPP content was raised. In a PBS solution with a pH of 4.5, the SF exhibited efficient release from the nanoparticles, demonstrating that the delivery mechanism is effective for tumour cells. The cytotoxicity investigation showed that their anticancer effect against HepG2 cell lines was significantly superior than that of free SF. In addition, the nanodrug demonstrated an absence of any detectable toxicity to normal adult human dermal fibroblast (HDFa) cell lines. This is a step towards developing a more effective anticancer medication delivery system with sustained-release characteristics, which will ultimately improve the way cancer is managed.

## Introduction

Macromolecular chemists successfully delivered anti-cancer therapy using nanomaterials, with a focus on the host–guest technique. The invention of nanodelivery devices containing therapeutic chemicals has increased the therapeutic efficacy of several cancer medications. Polymeric nanocarriers have recently been designed to efficiently load medications, release those drugs in a controlled and sustained manner, and accumulate enough pharmaceuticals at illness sites. Modern traditional cancer therapies, such as rigorous chemotherapy, radiation, surgery, and immunotherapy, are characterised by high toxicity, medication losses, injury to healthy organs or cells, non-specific distribution, and significant side effects, all of which contribute to patients' poor prognosis. Traditional treatments for liver cancer are plagued by issues such as drug loss, drug clearance, drug resistance, and drug accumulation in the tumour site. Therapeutic nanodelivery technologies provide several advantages over regular cancer treatment. Therapeutic delivery methods are critical in the clinic because medications must be loaded, encapsulated, efficiently taken up by cells, released slowly over time, and stored inside cancer cells. It is therefore vital to develop more efficient and effective nanocarrier drug delivery methods in order to transport the treatment to the specific affected areas or cancer cells with adequate concentrations of drug and an excellent therapeutic duration period. To combat the negative effects of chemotherapy that might manifest in the body, this is required.

The most common form of liver cancer, hepatocellular carcinoma (HCC), also known as hepatoma, accounts for 85 to 90% of cases^1^. As a result of the widespread spread of hepatitis B virus (HBV) in these regions, HCC is disproportionately prevalent in poorer nations. Unfortunately, both the overall mortality rate and the death rate specifically attributable to HCC are on the rise in industrialised nations. High-grade hepatocellular carcinoma caused by HCV is the fastest-growing cancer killer worldwide. Both public health organisations and clinical and fundamental biological researchers are always thinking about ways to actively curb the rising death rates owing to HCC. In order to reduce the likelihood of HCC developing in high-risk people, the following procedures can be taken:

I. Controlling risk factors or reducing the likelihood of those at risk developing head and neck cancer are the goals of the preventative measures referred to as HCC prevention measures.
II. Monitoring for early diagnosis of HCC at a treatable stage
III. Improving the management of individuals with HCC diagnosed through
  - Better patient classification for improved selection of therapy modalities to be provided to patients in order to improve cure and survival rates.
  - Introduction of innovative therapeutic approaches for the treatment of HCC

**Figure 1:**
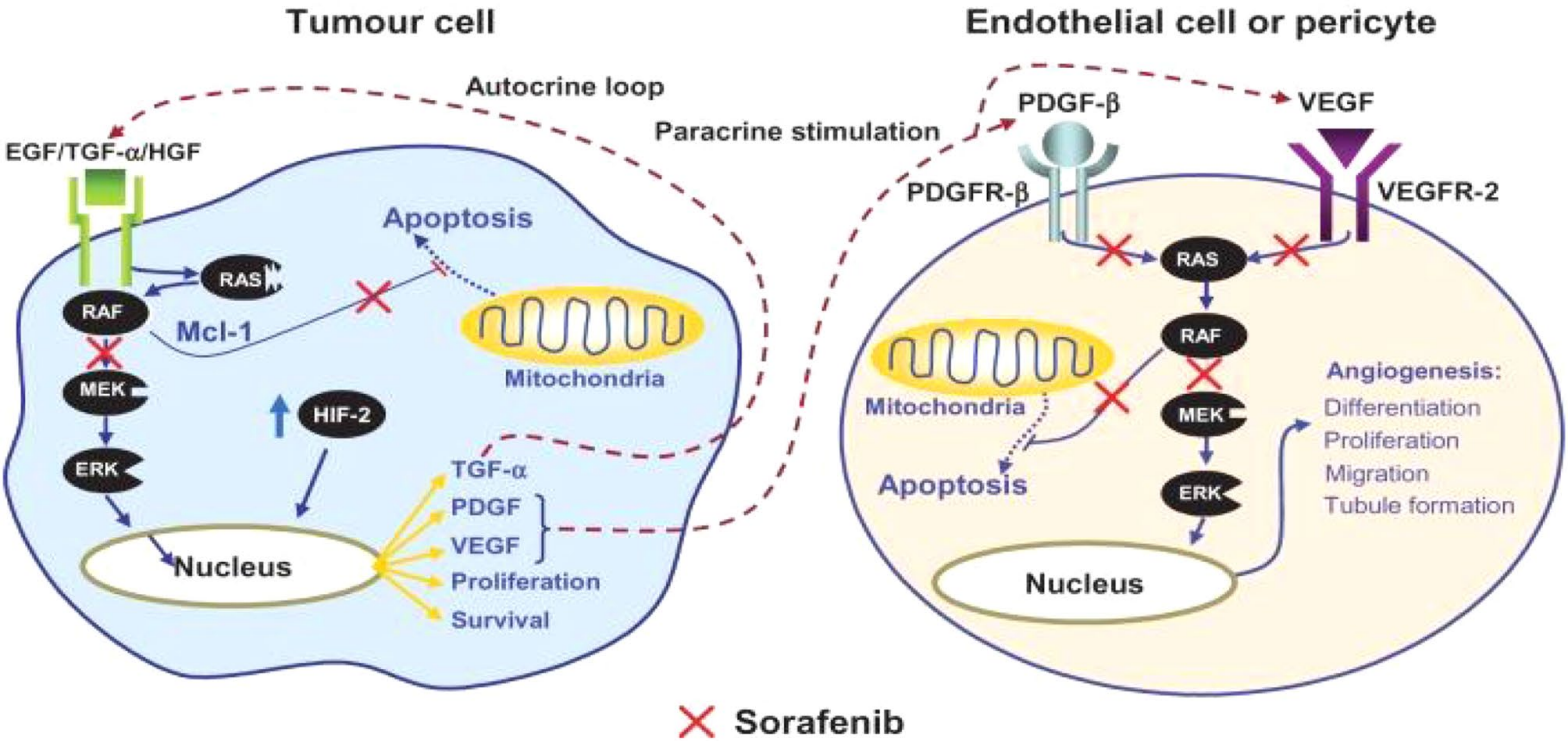
Mechanism action of SF for cancer treatment^7^.

In accordance to studies, SF is an orally given multi-kinase inhibitor that has significant anticancer effects via anti-proliferative, anti-angiogenic, and pro-apoptotic mechanisms^2^. Raf-1, B-Raf, VEGFR-1, VEGFR-2, VEGFR-3, platelet-derived growth factor receptor- (PDGFR-), FLT-3, Ret, and c-Kit are all downregulated. Raf protein has been found to be an important component of the RAS/MEK/ERK signalling cascade. The MAPK/ERK pathway is composed of three sequentially activated protein kinases that are crucial mediators of a variety of cellular fates, including growth, proliferation, and survival. On the other hand, SF injection was discovered to inhibit the activation of hypoxia-inducible factor-1 (HIF-1) protein^3,4^. Modulating anti-angiogenetic action is crucially dependent on HIF-1 protein. In addition, SF has successfully downregulated the expression of the anti-apoptotic myeloid cell leukaemia 1 (Mcl-1) protein, which may boost the pro-apoptotic therapeutic efficacy in combination therapy with other anti-cancer medications^5,6^. Figure 1 depicts an overview of the SF inhibition process.

Nanoparticles are defined as materials that possess dimensions within the nanoscale range, specifically below 100 nm. In recent times, these substances have gained significance in contemporary medicine, finding utility in diverse areas such as serving as contrast agents in medical imaging and as carriers for gene delivery into individual cells. Nanoparticles possess unique properties that differentiate them from bulk materials due to their size, including but not limited to chemical reactivity, energy absorption, and biological mobility. There are numerous benefits of nanoparticles to modern medicine. There are certain scenarios where the utilization of nanoparticles facilitates analyses and therapies that are otherwise unattainable.

To improve the anticancer effects of sorafenib medication to liver cancer cell lines, this work intends to optimise the formulation of SF–CS NPs by adjusting the quantity of TPP. Using the ionic-gelation technique, the phosphate group in TPP reacted with the amine group in chitosan to form crosslinks that allowed the synthesis of the SF–CS NPs. To optimise the therapeutic payload, the nanoparticles’ cross-linking characteristics between chitosan chains and their interaction with TPP can be adjusted. The efficacy of the resultant nanoparticle will be evaluated by physicochemical characterizations, in vitro release, and anticancer activity by MTT assay on liver cancer cell lines.

## Materials, methods, and characterizations

All chemicals were obtained from reputable suppliers: Sigma Aldrich (Saint Louis, MO, USA) provided chitosan powder [low molecular weight (LMW), deacetylation 75–85%], Merck supplied sodium tripolyphosphate (TPP), Hamburg Industries Inc. (Germany) supplied acetic acid (99.8%), and Laboratory & Scientific Enterprise (Malaysia) supplied dimethyl sulfoxide (99%). The Tween 80 and SF medications were purchased from Sigma-Aldrich. The entire experiment was conducted with deionized water (18.20 MΩ cm^−1^). Primary Dermal Fibroblast: Normal, Human, Adult (HDFa) (ATCC® PCS-201-012™), Hepatocellular carcinoma cell, (HepG2-ATCC® HB-8065™), were purchased from ATCC (AMERICAN TYPE CULTURE COLLECTION, PO BOX 1549, Manassas, VA 20,108, USA).

### Synthesis of SF–CS NPs

**Figure 2:**
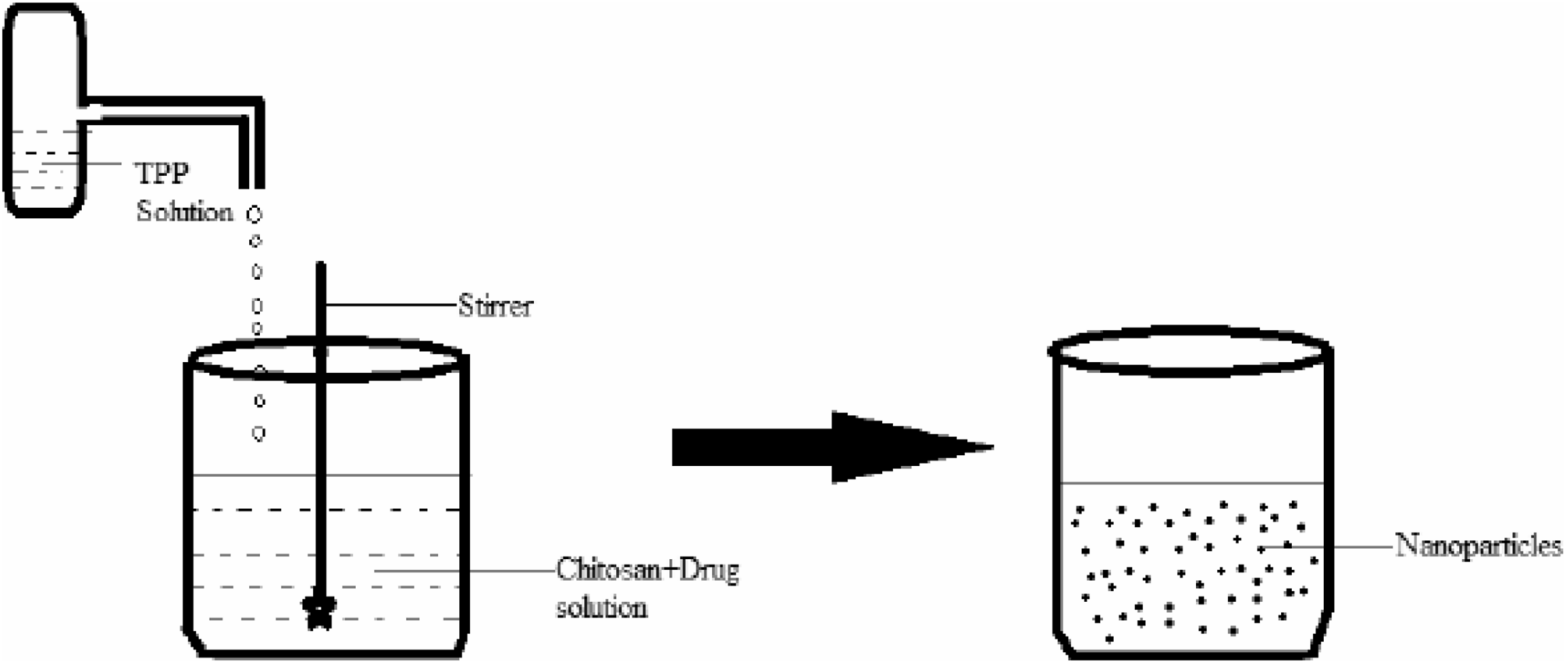
Schematic diagram on ionic gelation methodology ^10^.

Ionic gelation methodology was used to fabricate SF–CS NPs in this investigation (Fig. 2). LMW chitosan powder (0.5 mg/mL) in the 1.0% (v/v) acetic acid solution was completely dissolved^8,9^. Afterwards 1.0 g of SF medications were dissolved in 100 mL of DMSO. With constant stirring, drug solutions and TWEEN-80 surfactant at a concentration of 2.0% (v/v) were added into the aqueous solution containing the dissolved chitosan. Parallelly, a solution of TPP at concentrations of 2.5, 5.0, 10.0, and 20.0 mg/mL was prepared in water correspondingly. The TPP solution was added to the chitosan drug solution drop-by-drop using a burette. The suspension was centrifuged for 10 min at 4000 rpm, and then washed three times with water. To facilitate further analysis and cytotoxicity testing, the sample was freeze-dried.

### Instrumentation and characterizations

In this study, a nanosizer (Malvern Instruments, Malvern, UK) was utilised in order to ascertain the SF–CS NPs’ particle size distribution. High-resolution transmission electron microscopy (HRTEM; Hitachi H-7100, Tokyo, Japan) at 100 kV accelerating voltage was used to examine the morphological traits and size distribution. A programme for image processing known as ImageJ was used to assess the particle size distribution. A drop of diluted SF–CS NPs solutions was applied to copper grids with a mesh size of 300 and carbon films. The samples were air-dried prior to HRTEM analysis. The next step was conducting a thermogravimetric and differential thermogravimetric analysis (TGA/DTG) on the nanoparticles using a Mettler-Toledo apparatus between the temperatures of 25 and 1000 °C with a heating rate of 10 °C per minute. The geometry and morphology of SF–CS NPs nanoparticles were investigated utilising a field emission scanning electron microscope (FESEM, NOVA NANOSEM 230, NovaTM NanoSEM 230—FEI Corporation, CA, USA). The nanoparticle liquid solutions were placed on a stub, dried in an oven, and then examined^11,12^. Energy Dispersive X-Ray spectroscopy in combination with FESEM (FESEM-EDX, NOVA NANOSEM 230, NovaTM NanoSEM 230—FEI Corporation, CA, USA) was used to determine the composition of the produced SF–CS NPs^13^. The EDX spectrum was used to quantify the atomic and weight percentages of elements present in the sample not limit to oxygen, nitrogen, hydrogen, sulphur, and carbon compounds. To determine the loading efficiency and drug release of the SF–CS NPs, an ultraviolet–visible (UV–Vis) spectrophotometer was utilised. FTIR study of SF–CS NPs was performed using an FTIR spectrometer (PerkinElmer1725X spectrophotometer). FTIR spectra with the potassium bromide pellet (1.5 mg of sample + 150 mg of potassium bromide) were used to examine the successful construction of the nanoparticles. The resolution of the instrument was 4 cm^−1^. The range from 600 to 4000 cm^−1^ in the FTIR spectra was examined.

### Drug content determination

An efficiency curve for drug loading was produced using a wavelength range of 200–500 nm. In all, 1 mL of methanol was spiked with 10 mg of SF–CS NPs powder from each sample. The concentration of the medication was then calculated using UV–Vis spectroscopy and the peak wavelength of absorption. Using this approach, we were able to determine the encapsulation efficiency (EE%) and loading content (LC%) of SF–CS NPs.

1 $$EE\,\left( \% \right) = \frac{{\left[ {Total\;nanoparticle\;with\;drug - Free\;drugs} \right]}}{{\left[ {Total\;nanoparticle\;with\;drug} \right]}}\; \times \;100\%$$

2 $$LC\,\left( \% \right) = \frac{{\left[ {Total\;nanoparticle\;with\;drug - Free\;drugs} \right]}}{{\left[ {Total\;nanoparticle\;with\;drug} \right]}} \times 100\%$$

### In vitro drug release

Synthesized nanoparticles (SF–CS NPs) released drugs in a manner similar to, and in some aspects identical to, the method described in a paper by Jasim and coworkers (2022). To resuspend the drug-loaded nanoparticles (10 mg), pH 7.4 and 4.8 phosphate-buffered solution (PBS) was used at 37 °C with moderate and continuous shaking at 400 rpm using an orbital shaker. Finally, 3 mL aliquots of the supernatant were taken from the centrifuged samples at predetermined intervals (0.5, 1, 2, 4, 6, 12 and 24 h, and later every 24 h up to 7 days). They used fresh PBS solution to refill the syringe. UV–Vis spectrophotometry was used to examine the wasted supernatant. As a matter of fact, three different measurements were taken.

The drug release from the SF–CS NPs was based on, and in some ways identical to, the approach published by Jasim et al. (2022). Ten milligram of SF–CS NPs were resuspended in 10 mL of pH 7.4 and 4.8 phosphate-buffered solution (PBS) at 37 °C, with moderate and continuous shaking at 400 rpm using an orbital shaker. Thereafter, at regular intervals, 3 mL aliquots of the supernatant were collected from the centrifuged samples (0.5, 1, 2, 4, 6, 12 and 24 h, and later every 24 h up to 7 days). They refilled the syringe with a new PBS solution. Analysis of the discarded supernatant was performed using UV–Vis spectrophotometry. There were three separate sets of measurements taken.

### In vitro cell viability assay

Cell survival and cytotoxicity of SF–CS NPs were evaluated in a number of different cell lines using the methyl thiazole tetrazolium (MTT) assay^14,15^. This study used the HepG2 cell line as well as the HDFa cell line to assess the toxicity of the nanoparticles. HepG2 cells were cultured in Eagle's Minimum Essential Medium (EMEM) supplemented with 10% foetal bovine serum (FBS) and 1% penicillin–streptomycin (PS; 100 U/mL), whereas HDFa cells were grown in fibroblast basal medium with Fibroblast Growth Kit-Serum-Free (ATCC® PCS-201-040). At 80% confluence, the cells were washed three times with phosphate-buffered saline. Using trypsin at a concentration of 0.1%, the cell layers were harvested. SF–CS NPs were added to 96-well culture plates containing cells that had been seeded at a density of 1 × 10^4^ cells per well. The plates were then placed in an incubator for 24 h. These are the steps taken to calculate the relative cell viability percentage:

3 $$\% \;of\;cell\;viability = \frac{{\left( {A_{treatment} - A_{blank} } \right)}}{{\left( {A_{control} - A_{blank} } \right)}} \times 100\% \;\left( {{\text{where}},\;{\text{A}} = {\text{absorbance}}} \right).$$

## Results and discussion

### Reaction yield, encapsulation efficiency and loading content

The maximum yield of the reaction was recorded at 5 mg/mL of TPP, as shown in Table 1, and even after increasing the concentration of TPP, the yield remained relatively similar. At a concentration of 2.5 mg/mL, the reaction yield was lowest. The findings of loading content (LC) and encapsulation efficiency (EE) of SF showed no definite pattern when TPP concentrations were increased. The LC and EE achieved a maximum concentration of 5 mg/mL but declined at 10 and 20 mg/mL TPP. This may be because to the lower nanoparticle sizes of SF–CS NPs with a 10 mg/mL and 20 mg/mL TPP concentration, SF–CS NPs10 and SF–CS NPs20 (the sizes of the nanoparticles are deliberated later).

**Table 1:** Evaluation of SF–CS NPs’ encapsulation effectiveness, loading material quantity, and reaction yield in numerical form.

Nanoparticles	Reaction yield (%)	Loading content (%)	Encapsulation efficiency (%)
SF–CS NPs 2.5	79.5 ± 4.5	14.16 ± 1.5	58.7 ± 1.1
SF–CS NPs 5.0	83.3 ± 3.0	20.54 ± 1.5	70.2 ± 1.6
SF–CS NPs 10	82.4 ± 1.5	17.56 ± 2.1	68.8 ± 2.8
SF–CS NPs NPs 20	81.9 ± 1.5	17.12 ± 1.1	69.8 ± 3.5

### Morphological studies using the HRTEM

**Figure 3:**
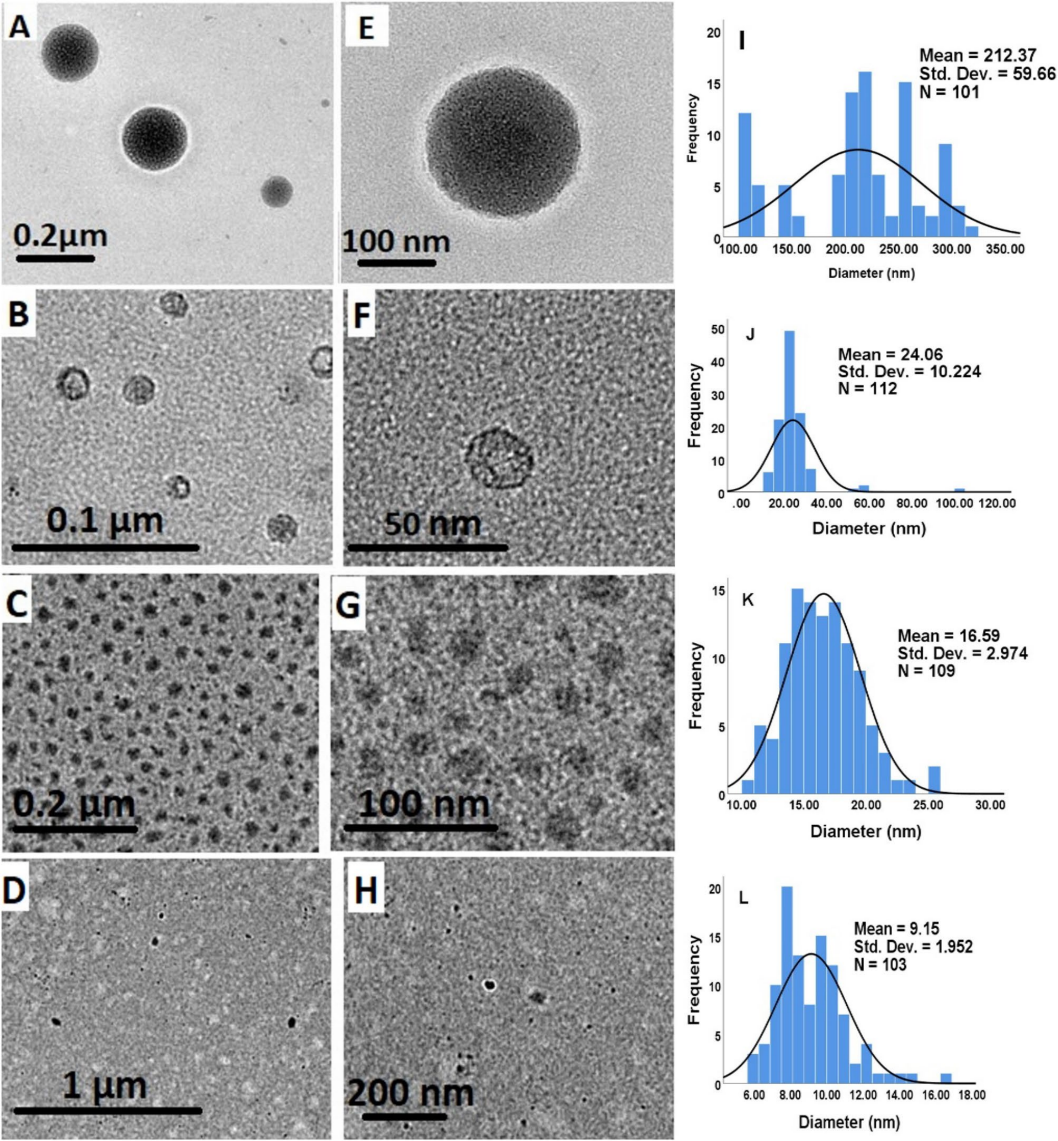
HRTEM images and particle size distribution of (**A**, **E**) SF–CS NPs 2.5, (**B**, **F**) SF–CS NPs 5.0, (**C**, **G**) SF–CS NPs 10 and (**D**, **H**) SF–CS NPs 20.

The morphology of the particles was deduced from HRTEM images by examining the nanoparticles’ precise diameter, size distributions, shape, and interface image of the particles' dispersion in the solution. Each of the produced nanoparticles took on the form of a spherical, as seen in Fig. 3. Also, it was possible to see how the concentration of TPP affected the results, with a decreasing mean diameter size as the concentration of TPP grew. At 2.5 mg/mL, the lowest concentration, SF–CS NPs 2.5 displayed the relatively biggest spherical particle with a mean diameter of 212.4 ± 59.7 nm, followed by SF–CS NPs 5 and SF–CS NPs 10 (5 and 10 mg/mL TPP) with mean diameters of 24.1 ± 10.2 and 16.6 ± 3.0 nm, respectively. The SF–CS NPs 20 also demonstrated the comparatively lowest spherical particle size with a mean diameter of 9.2 ± 2.0 nm at the maximum TPP concentration of 20 mg/mL.

### Particle size distribution analysis

**Figure 4:**
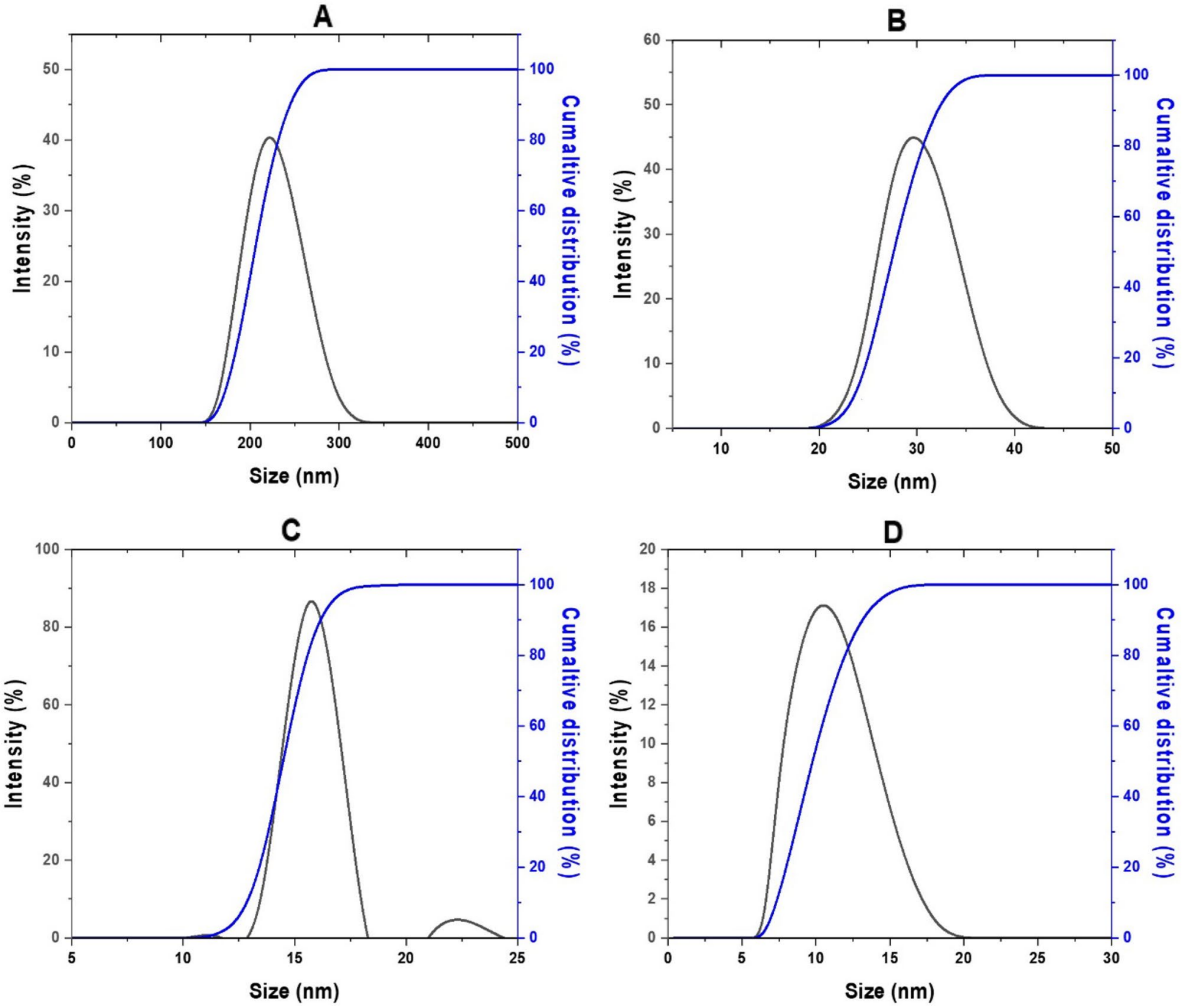
Particle size distribution of SF–CS NPs ready at numerous concentrations of TPP, (**A**) 2.5, (**B**) 5, (**C**) 10 and (**D**) 20 mg/mL.

The particle size distribution was also investigated in the solvated condition, where molecules of the solvent (deionized water) interacted with the particles. Figure 4 depicts a similar pattern, in which raising the TPP concentration resulted in a decrease in the size of the generated nanoparticles, most likely due to adsorption of ions with opposite charges in the solvent medium (deionized water). It is well established that the free amino groups in chitosan interact with the negative charge of the multivalent anion, TPP, to create CS-TPP nanoparticles in an acidic environment. This process converts –NH_2_ to –NH^3+^. Henceforth, more inter- and intramolecular crosslinking between chitosan and TPP led to lower particle sizes as TPP concentration was raised.

### Surface properties using the FESEM with EDX

**Figure 5:**
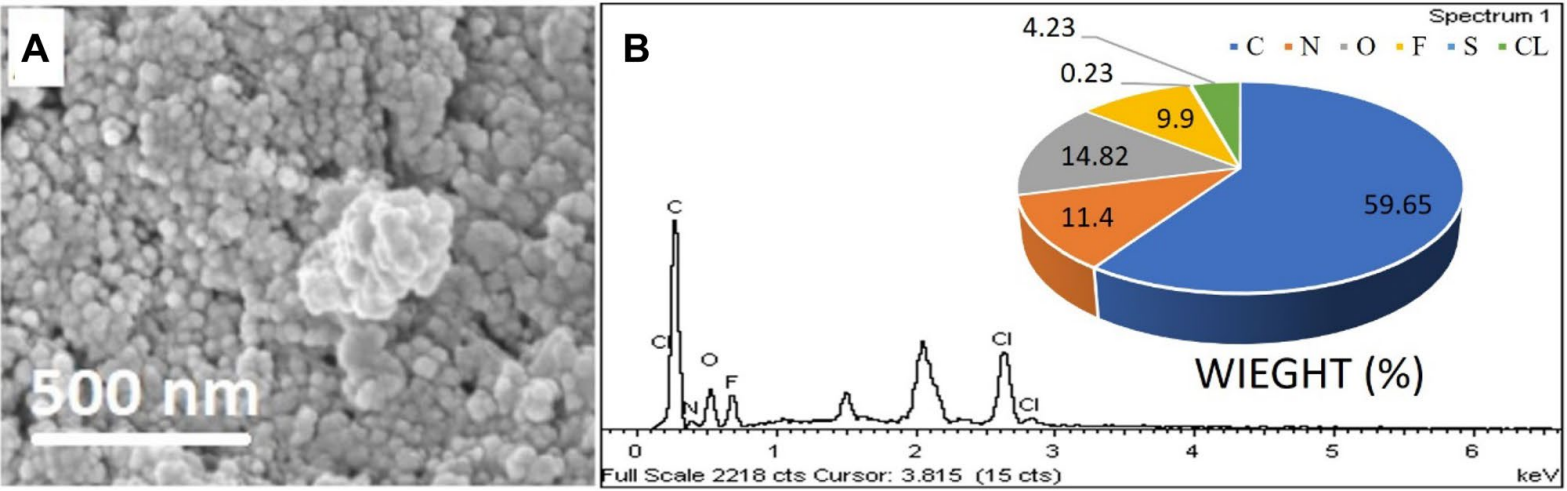
FESEM images of SF–CS NPs 5.0 in conjunction with EDX spectra.

The surface morphology of the synthesized nanoparticles was observed by the FESEM (Fig. 5). EDX analysis in conjunction with FESEM was conducted to investigate the compositional analysis of SF–CS NPs 5.0. A FESEM analysis of the SF–CS NPs 5.0 confirmed the spherical nature of the nanoparticles. Apart from that, the inclusion of C, N, O, F, S, and Cl elements is demonstrated in the Fig. 5, proving that all compounds were included in the final nanoparticles, SF–CS NPs 5.0. In terms of atomic percentages and molecular weights, carbon and oxygen were found to be the most prevalent, whereas nitrogen, fluorine, sulfur and chlorine were found to be present due to the presence of the medications. The observed Cl peak can be attributed to the presence of SF.

### X-ray diffraction

**Figure 6:**
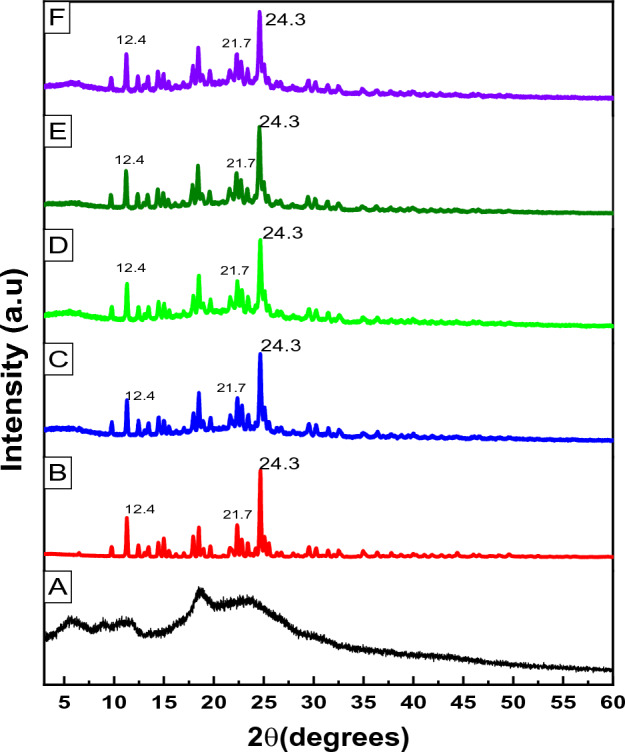
XRD patterns of (**A**) CS NPs (**B**) pure SF, (**C**) SF–CS NPs 2.5, (**D**) SF–CS NPs 5.0, (**E**) SF–CS NPs 10 and (**F**) SF–CS NPs 20.

Figure 6 displays the XRD patterns pure SF, CS NPs and formed SF–CS NPs with varied TPP concentrations. Pure SF had a strong reflection at 2θ = 25.1°, as seen in Fig. 6, indicating it is extremely crystalline in nature. CS NPs, on the other hand, had a wide peak, indicating that it is an amorphous sort of substance. A large amorphous peak was seen for the nanoparticles SF–CS NPs 2.5, SF–CS NPs 5.0, SF–CS NPs 10, and SF–CS NPs 20, indicating a high concentration of the chitosan phase, behind which the crystalline peak of SF was buried when they were enclosed in the CS NPs. Broad peaks at diffraction angles 2θ of 12.4, 21.7 and 24.3° matched the peak pattern of pure SF, indicating that SF had been encapsulated in the chitosan matrix.

### Fourier-transform infrared

**Figure 7:**
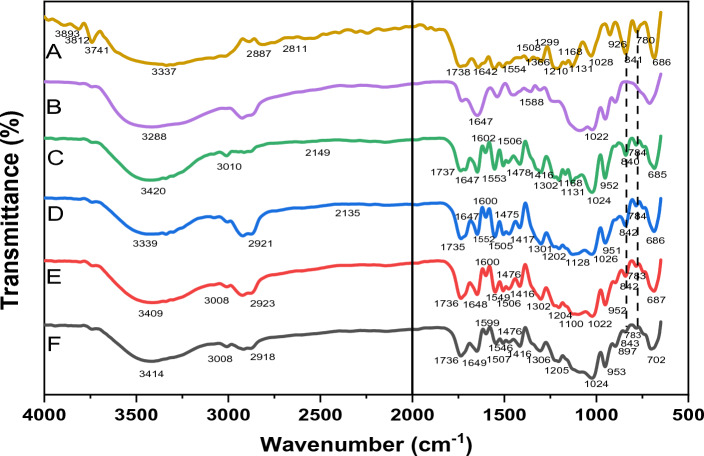
FTIR spectrum of (**A**) pure SF, (**B**) CS NPs, (**C**) SF–CS NPs 2.5, (**D**) SF–CS NPs 5.0, (**E**) SF–CS NPs 10, and (**F**) SF–CS NPs 20.

Figure 7 displays the Fourier-transform Infrared (FTIR) spectra for pure SF, CS NPs, SF–CS NPs 2.5, SF–CS NPs 5.0, SF–CS NPs 10, and SF–CS NPs 20, which may be used to infer the presence and interaction of adsorbed molecules and coated molecules bonded to the surface layers on the nanoparticles. The absorption bands were detected by scanning a spectrum between 600 and 4000 cm^−1^.

For pure SF, the peak at 1642 cm^−1^ is characteristic of the C=O amide group. The C–H stretching band has a companion band at 3078 cm^−1^, and the amide C=O group has a characteristic band at 1738 cm^−1^. The band at 1022 cm^−1^ was, on the other hand, caused by the phosphate group in TTP. Moreover, CS NPs had characteristic broad bands at 1647 and 1588 cm^−1^, which were interpreted to reflect the stretching of the CO–NH_2_ group bending vibration. The C–O group of the carboxylic acid causes a band to appear at 1302 cm^−1^ and 1128 cm^−1^. Nanoparticles with the SF–CS NPs 2.5, SF–CS NPs 5.0, SF–CS NPs 10, and SF–CS NPs 20 produced had bands at 3332, 3294, 1704, 1641, 1556, and 1202 cm^−1^. The SF–CS NPs 679 cm^−1^ band is a result of the drug’s C–Cl stretching. In addition, the bending vibration of the CO–NH_2_ group was shown to be stretched in ChNPs alone, as indicated by broad bands at 1647 and 1588 cm^−1^. All the manufactured nanoparticles displayed the CS-TTP signature bands, proving that the SF had been successfully encapsulated within the chitosan matrix.

### Thermal analyses

**Figure 8:**
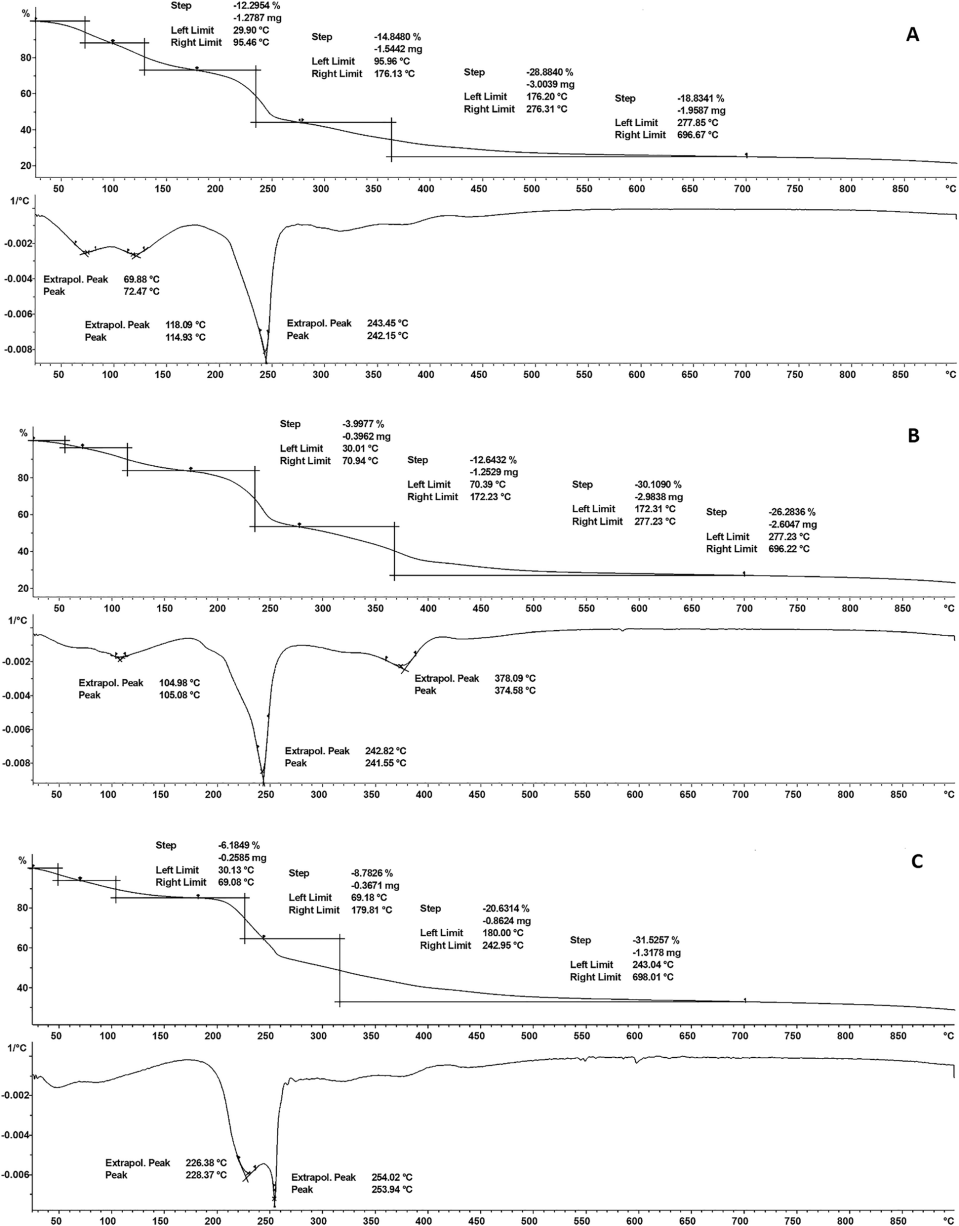

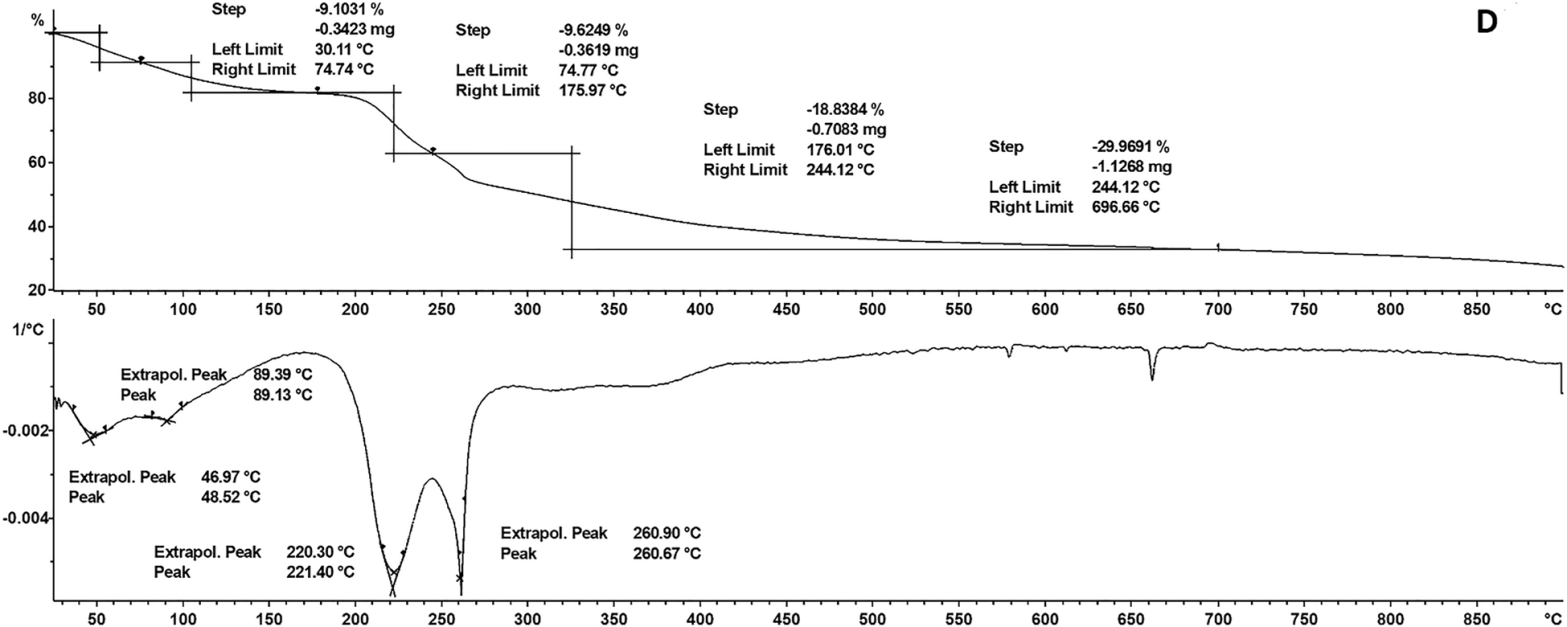
TGA/DTG thermograms of (**A**) SF–CS NPs 2.5, (**B**) SF–CS NPs 5.0, (**C**) SF–CS NPs 10 and (**D**) SF–CS NPs 20.

Using a thermal analyzer, the thermal stability of the SF–CS NPs 2.5, 5.0, 10, and 20 was investigated. The results TGA/DTG thermograms are shown in Fig. 8. The results provide quantitative details on the composition of the nanostructures. The SF–CS NPs 2.5, SF–CS NPs 5.0, SF–CS NPs 10, and SF–CS NPs 20 revealed comparable four-phase weight loss patterns, as shown in Fig. 8. Weight loss at temperatures between 28 and 100 °C was attributed to the release of water (loss of hydrogen bonds). In contrast to the third and fourth phases, which occurred between 69 and 180 °C, the second stage was triggered by the breakdown of chitosan. The final residue from the TGA study ranged between 20 and 30 percent demonstrating the higher thermal stability of chitosan.

### In vitro drug release study

**Figure 9:**
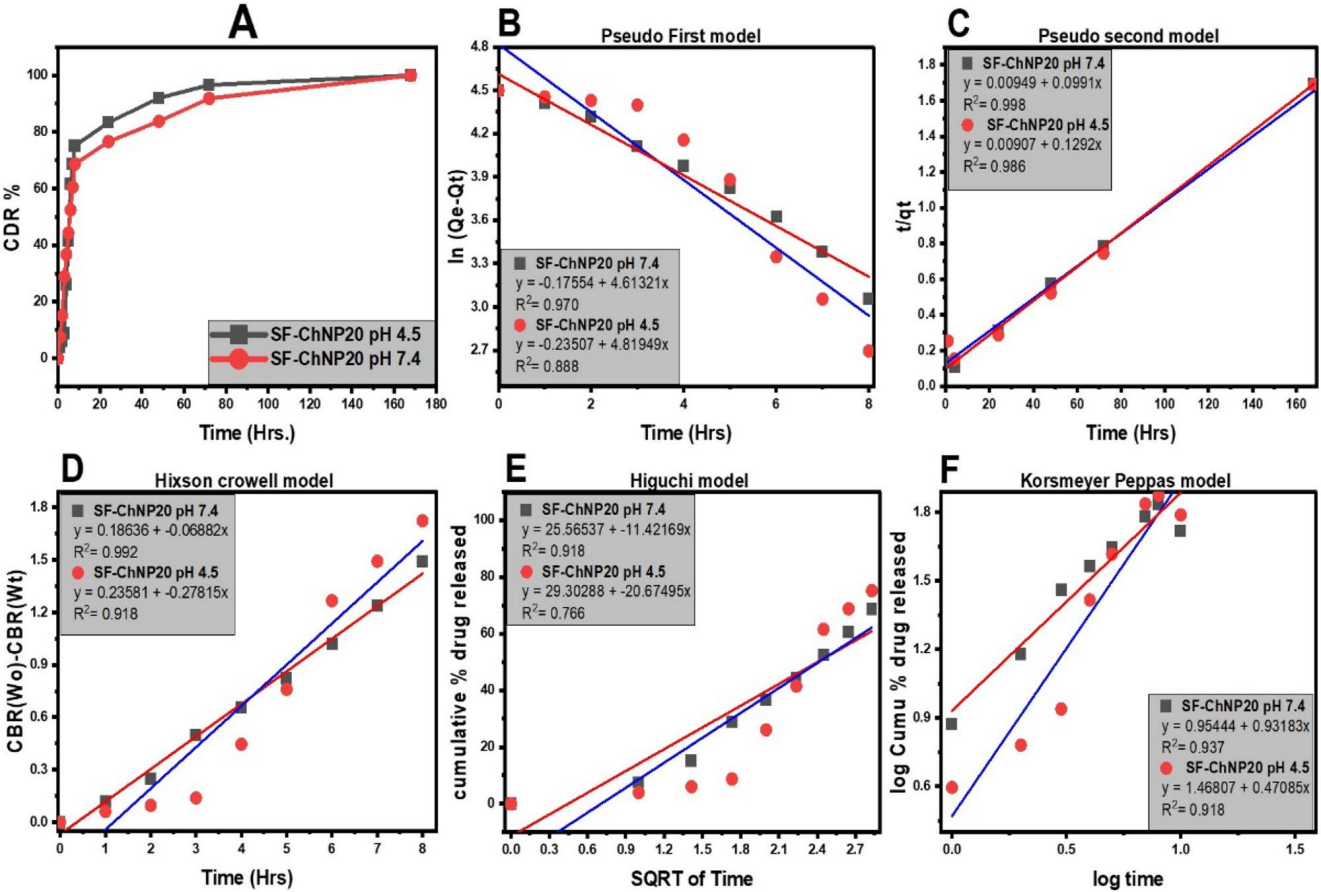
Cumulative release profiles of SF from (**A**) SF–CS NPs 5 and SF–CS NPs 20 at pH 4.5 and (**B**–**F**) the fitting of the data using five different mathematical models at various pH 4.5.

The therapeutic effectiveness of the medication greatly depends on the type of the drug release from the nanocarrier, which must be maintained for the delivery system to be effective after administration. Controlled drug delivery has the potential to reduce medication dosages, minimise systemic adverse effects, and lengthen the time when therapeutic medicines are effective. The amount of SF released from SF–CS NPs 20 was measured using in vitro release assays in PBS 7.4 and PBS pH 4.5. For the simulation of the physiological conditions of cells and the acidic tumour microenvironment, pH values of 4.5 and 7.4 were used, respectively. Same stages were followed by SF–CS NPs 20, who first released quickly for the first 24 h, then slowly over the next 48 h, and eventually steadily. The constant release of SF that SF–CS NPs 20 display in Fig. 9A suggests that SF is stable inside the nanoparticles. The 169-h period ended with a successful complete discharge. Figure 9 shows that 92% of the SF released after 72 h was achieved at a pH of 4.5 as contrasted to 84% at a pH of 7.4 for the SF–CS NPs 20 samples at the same time point. The 169-h period ended with a successful complete discharge. These release profiles revealed that SF–CS NPs 20 may release SF at pH 4.5 rather than pH 7.4, which may make SF more susceptible to release.

Due to the numerous physicochemical variables involved in medication administration or release from nanocarriers, mathematical models are required. Figure 9B illustrates the linear fits to five alternative kinetics and mathematical models that were utilised to fit the data of SF release from nanoparticles (F). Formula 4, in where K_1_ is the rate constant and qe and q_t_ are the quantities of SF released at equilibrium and at any time (t), respectively, may be used to define pseudo-first-order release kinetics in a linear form. The rate constant, K_2_, for pseudo-second-order release kinetics is described by Formula 5 in a linear form typical of second-order kinetics models. According to the Higuchi model (Formula 6), where K_H_ is the Higuchi rate constant, the sorafenib release from the nanoparticles rises with the square root of time. Formula 7 from the Hixson–Crowell model illustrates the time dependency of the residual SF cube root, where M_o_ is the initial concentration of SF in the nanoparticles, q_t_ is the quantity released at time t, and K_HC_ is the Hixson–Crowell rate constant. The release exponent n and the logarithm of time are related in the Korsmeyer–Peppas model (Formula 8), where q represents the release at infinite time.

The calculated correlation coefficient (R_2_), rate constant (K), and t_1/2_ values of the release profile are shown in Table 2, demonstrating that the pseudo-second-order kinetics model was followed by the release kinetics of SF–CS NPs 20. It was discovered via the development of competing exchange kinetics that the ionic reaction is the rate-determining stage in the releasing process.

4 $$In(q_{e} - q_{t} ) = In(q_{e} - K_{1} t)$$

5 $$\frac{t}{{q_{e} }} = \frac{1}{{K_{2} q_{e}^{2} }} + \frac{1}{{q_{e} }}$$

6 $$q_{t} = K_{H} \sqrt t$$

7 $$\sqrt[3]{{M_{0} }} - \sqrt[3]{{q_{t} }} = K_{HC} t$$

8 $$q_{t} lq_{\infty } = Kt^{n}$$

**Table 2:** The drug release data from the SF–CS NPs 20 in PBS solution pH 4.5 and 7.4 were fitted using the correlation coefficients (R_2_) and rate constants (K).

The pH of release media	Material	Saturation release (%)	Pseudo-first-order		Higuchi model		Pseudo-second-order		Hixson–Crowell model		Korsmeyer–Peppas model		t_1/2_ (h)
			R^2^	K_1_ (ln mg h^−1^)	R^2^	KH (mg√h^−1^)	R^2^	K_2_ (mg h^−1^)	R^2^	K_HC_ (h^−1^)	R^2^	K (h^−1^)	
4.5	SF–CS NPs 20	98.89	0.888	− 0.23507	0.766	29.30288	0.986	0.00907	0.918	0.23581	0.918	1.46807	76.40
7.4		95.78	0.970	− 0.17554	0.918	25.56537	0.998	0.00949	0.992	0.18636	0.937	0.95444	73.024

### In vitro bioassay

The smallest nanoparticles had the best antiproliferative activity and selectivity for HepG2 cells. The sole cytotoxicity and antiproliferative activities of SF–CS NPs 20 were therefore included in this section. The MTT test was employed to assess the cytotoxicity and cell survival levels of the SF–CS NPs 20. HDFa and HepG2 cells were used to perform this assay. For the purpose of determining tumour selectivity, HDFa cells were used as a non-cancerous cell line and a control.

**Figure 10:**
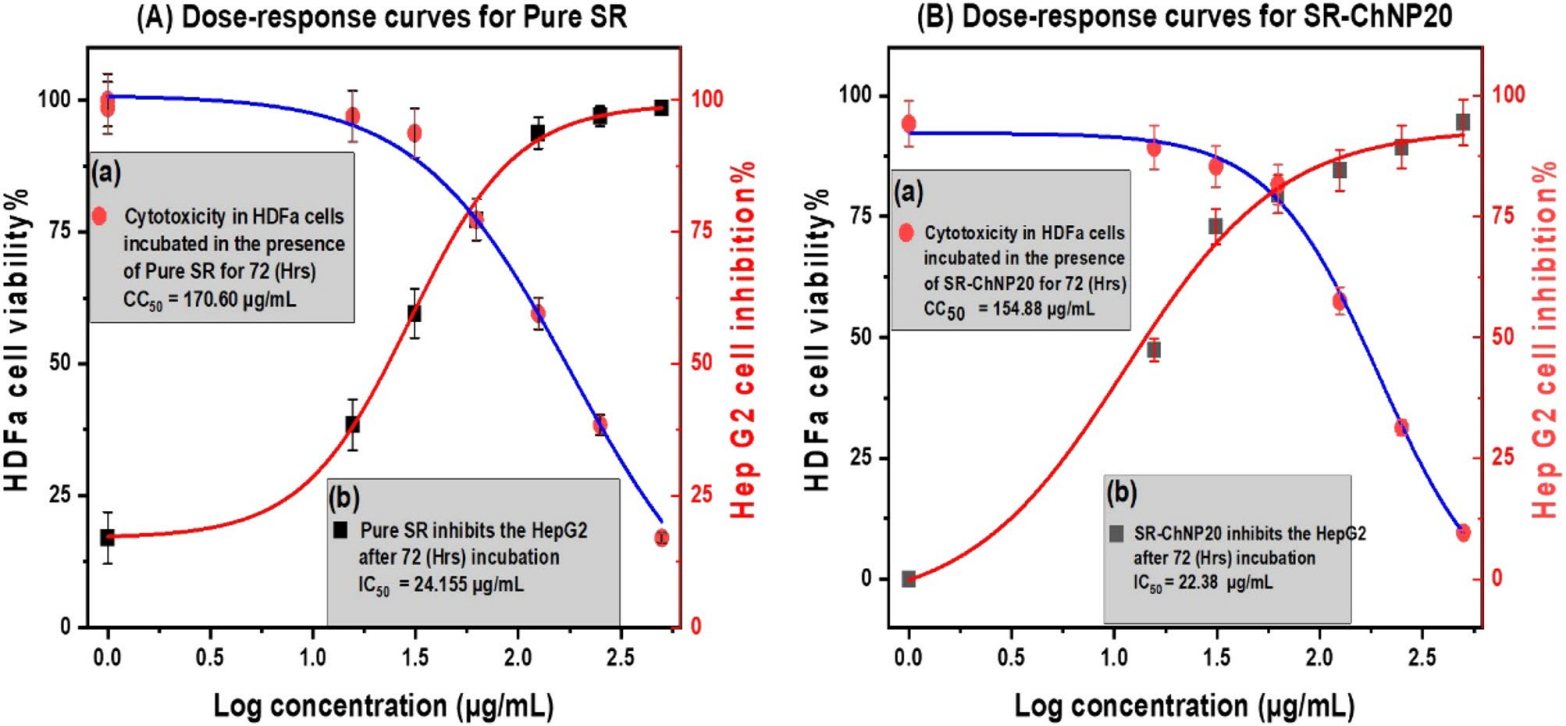
Dose–response curve of the (**A**) pure SF and (**B**) SF–CS NPs 20, where (a) represent HDFa viability and (b) represent Hep G2 inhibition. Results are presented as the mean ± SD from three separate studies, each of which was conducted in triplicate.

After being exposed to pure SF and SF–CS NPs 20 throughout time, Fig. 10 shows the percentage of HDFa and HepG2 cells that are still alive. Following incubation, all modified nanoparticles were shown to be biocompatible and nontoxic to HDFa and HepG2 cells, with cell viability of 85% and 50%, respectively, throughout a concentration range of 1 g to 125 g. As healthy cells are anticipated to be unaffected by the synthetic anticancer nanoparticle composition, it is predicted to effectively destroy malignant cells while being safe for healthy cells.

### Dose–response curve and cytotoxicity

The CC_50_ was calculated using sigmoidal dose–response curves, and the results were described in Table 3 in this work. Cytotoxicity studies of pure SF and SF–CS NPs 20 were carried out extensively in vitro. For HDFa and HepG2 cells, 154.88 1.8 and 13.60 1.6 g/mL of SF–CS NPs 20 were required, respectively, to demonstrate the CC_50_. Whereas pure SF required a higher dose to have a 50% cytotoxicity impact, SF–CS NPs 20 only needed a lower dose to achieve the same effect. Additionally, this research showed that free was found to be less dangerous than SF–CS NPs 20.

**Table 3:** The 50% cytotoxic concentration CC_50_ of pure SF and SF–CS NPs 20 against HepG2 and HDFa cells.

Materials	HDFa		Hep G2	
	IC_50_ µg/mL	CC_50_ µg/mL	IC_50_ µg/mL	CC_50_ µg/mL
Pure SF	51 ± 3*	171 ± 3*	24 ± 1*	31 ± 1*
SF–CS NPs 20	151 ± 3*	155 ± 2*	22 ± 4*	14 ± 2*

*The values are mean ± SD of three independent experiments performed in triplicate. *p* < 0.05 compared to the control and the vehicle-treated group.

## Conclusion

In this study, various different methods of characterisation, as well as in vitro release and cell viability research, were utilised in order to conduct a comprehensive investigation into the characteristics of SF–CS NPs 20. Research using FTIR and XRD lends credence to the notion that nanoparticles may be produced. The size of the scattered particles was demonstrated using images from both HRTEM and FESEM. There was no evidence of agglomeration. It was shown that the medication release profile was beneficial for the treatment of nanoparticles. At the same time point, the anticancer impact of the produced nanoparticles was more potent than that of the free drug, which was in conformity with the results of the cytotoxicity assays.

In future, it is imperative to conduct a thorough investigation of the biological activities through quantitative uptake validations. Additionally, the utilization of in vivo animal models is deemed necessary and will constitute a significant aspect of our forthcoming research.

## Data Availability

All data generated or analysed during this study are included in this published article.
